# Individual differences in GHB consumption in a new voluntary GHB self-administration model in outbred rats

**DOI:** 10.1007/s00213-024-06537-5

**Published:** 2024-02-09

**Authors:** Casper J. H. Wolf, Marcia Spoelder, Harmen Beurmanjer, Ronald Bulthuis, Arnt F. A. Schellekens, Judith R. Homberg

**Affiliations:** 1 Donders Institute for Brain, Department of Psychiatry, Radboudumc, Cognition, and Behaviour, Reinier Postlaan 4, 6525 GC Nijmegen, The Netherlands; 2 Donders Institute for Brain Cognition and Behaviour, Department of Cognitive Neuroscience, Radboudumc, 6525 EN Nijmegen, The Netherlands; 3 Nijmegen Institute for Scientist-Practitioners in Addiction (NISPA), 6525 HR Nijmegen, The Netherlands; 4 Department of Primary and Community Care, Radboud University Medical Center, 6500 HB Nijmegen, The Netherlands; 5 Behavioural Science Institute, Radboud University, 6525 GD Nijmegen, The Netherlands; 6 Novadic-Kentron Addiction Care, 5261 LX Vught, The Netherlands; 7 Metris B.V, 2132 NG Hoofddorp, The Netherlands

**Keywords:** Gamma-hydroxybutyric acid, GHB, Drug addiction, Self-administration model, GHB use disorder, Substance use

## Abstract

**Background and purpose:**

The use of the recreational drug gamma-hydroxybutyric acid (GHB) has increased over the past decade, concomitantly leading to a higher incidence of GHB use disorder. Evidence-based treatment interventions are hardly available and cognitive effects of long-term GHB use remain elusive. In order to study the development of GUD and the causal effects of chronic GHB consumption, a GHB self-administration model is required.

**Experimental approach:**

Long Evans rats had access to GHB in their home cage according to a two-bottle choice procedure for 3 months. Intoxication and withdrawal symptoms were assessed using an automated sensor-based setup for longitudinal behavioral monitoring. Rats were trained in an operant environment according to a fixed ratio (FR) 1, 2, and 4 schedule of reinforcement. Addiction-like behaviors were assessed through progressive ratio-, non-reinforced-, and quinine-adulterated operant tests. In addition, the novel object recognition test and elevated plus maze test were performed before and after GHB self-administration to assess memory performance and anxiety-like behavior, respectively.

**Key results:**

All rats consumed pharmacologically relevant levels of GHB in their home cage, and their intake remained stable over a period of 3 months. No clear withdrawal symptoms were observed following abstinence. Responding under operant conditions was characterized by strong inter-individual differences, where only a subset of rats showed high motivation for GHB, habitual GHB-seeking, and/or continued responding for GHB despite an aversive taste. Male rats showed a reduction in long-term memory performance 3 months after home-cage GHB self-administration. Anxiety-like behavior was not affected by GHB self-administration.

**Conclusion and implications:**

The GHB self-administration model was able to reflect individual susceptibility for addiction-like behavior. The reduction in long-term memory performance upon GHB self-administration calls for further research into the cognitive effects of chronic GHB use in humans.

**Supplementary Information:**

The online version contains supplementary material available at 10.1007/s00213-024-06537-5.

## Introduction

Gamma-hydroxybutyric acid (GHB) is a neurotransmitter and a precursor and metabolite of the main inhibitory neurotransmitter gamma-aminobutyric acid (GABA). Next to its use as a pharmaceutical drug for narcolepsy and patients with an alcohol use disorder (AUD) (Busardò et al. 2015), GHB is increasingly being used as a recreational drug for its disinhibiting and anxiolytic effects. The majority of GHB users consume GHB recreationally, without adverse effects (Dijkstra et al. 2021). However, due to its addictive properties, 4–21% of frequent users develop GHB use disorder (GUD) (Dijkstra et al. 2021). GUD is characterized by frequent overdosing and comas, a severe and erratic withdrawal syndrome, and high relapse rates (Kamal et al. 2017).

GUD has been associated with (verbal) short-term memory deficits (Beurmanjer et al. 2022; Pereira et al. 2018). In addition, patients with GUD often experience comorbid anxiety- and mood-related symptoms (Beurmanjer et al. 2019). It is difficult to determine whether these cognitive and emotional problems are pre-existing (and increase GUD susceptibility), or whether these symptoms are caused by chronic GHB use. Animal studies provide the opportunity to study these relationships and its causality in an experimental model. The majority of previous pre-clinical GHB studies primarily studied the effects of acute and forced, non-voluntary GHB administration on neurotoxicity and cognition (Chen et al. 2017; Johansson et al. 2014; Kueh et al. 2008; Laraway et al. 2008; Sircar and Basak 2004). In contrast to the established preclinical alcohol- and cocaine self-administration models, a model for prolonged voluntary GHB self-administration is currently lacking.

The establishment of a GHB self-administration model is crucial to assess the causes and consequences of GUD, to understand the neurotoxic effects of GHB, and to eventually examine novel pharmacological interventions to, e.g., reduce relapse rates. Previous studies have shown that GHB administration induces rewarding effects in rodents (Itzhak and Ali 2002; Martellotta et al. 1997; Watson et al. 2010), allowing for the development of a GHB self-administration model. Colombo et al. (1995) initially demonstrated home-cage binge-like GHB consumption in outbred rats (Colombo et al. 1995). GHB self-administration has further been examined in ethanol-preferring rats, leading to increased GHB intake compared to outbred rats (Colombo et al. 1998). In mice, voluntary intravenous GHB self-administration for 14 days leads to an initial increase in GHB intake, followed by a subsequent decrease (Watson et al. 2010). However, it is unknown to what extent these paradigms serve as a model for GUD, since extensive characterization is lacking.

The aim of the current study was to develop an animal model for prolonged voluntary home-cage and operant GHB self-administration to examine potential GUD-like characteristics. We first performed a pilot study in rats, in which we determined the optimal GHB self-administration parameters and assessed whether (accidental) GHB overdosing occurred in the rats. Specifically, we (1) examined GHB intake patterns in a home-cage environment, (2) characterized the behavioral phenotype during GHB intoxication and withdrawal, and (3) tested multiple addiction-like behaviors in an operant setting. In addition, we explored the predictive effect of baseline anxiety-like behavior on GHB intake, and assessed the effects of chronic GHB self-administration on cognition and anxiety-like behavior.

## Methods

**Fig. 1:**
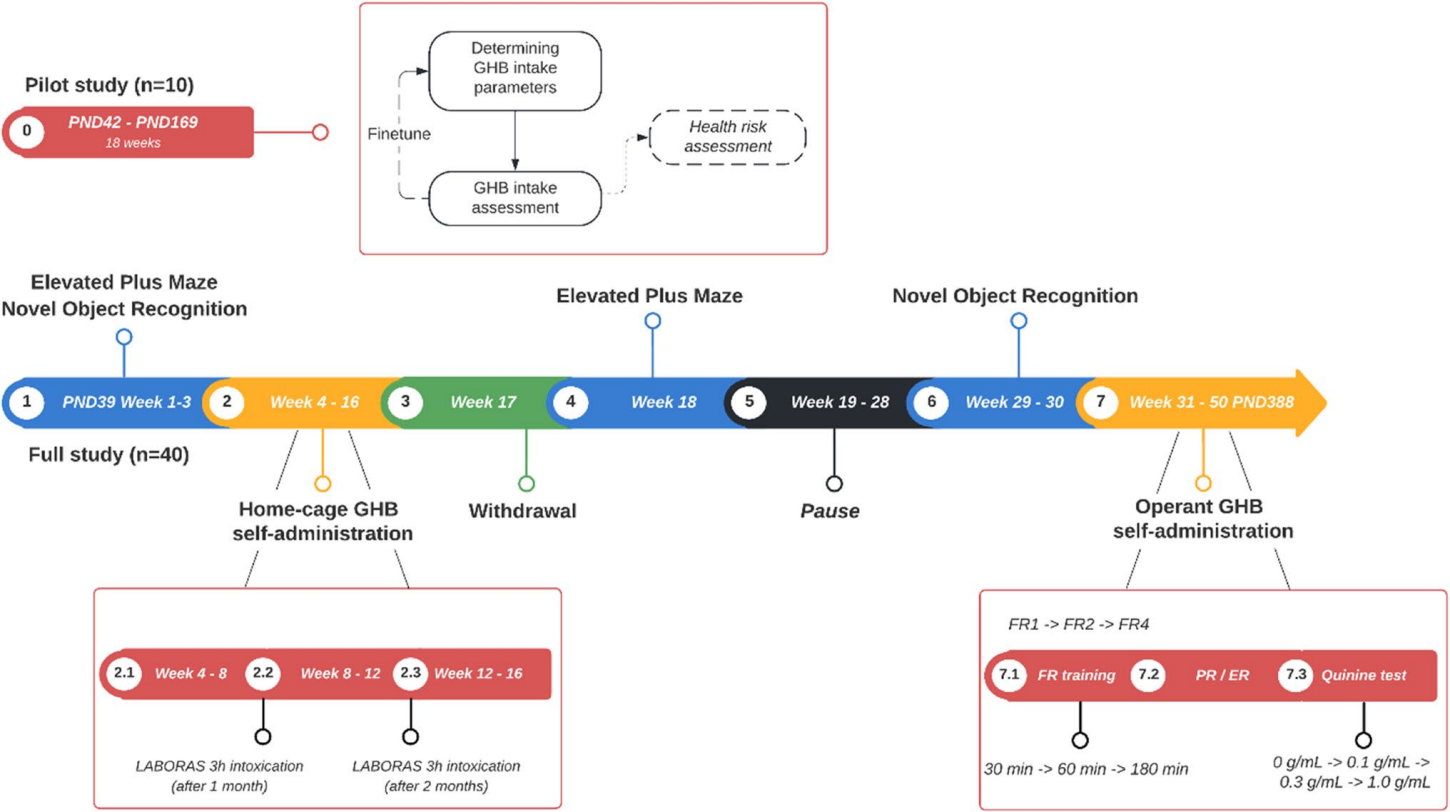
Overview and timeline of the experimental procedures that were performed in this study. Behavioral tests are represented in blue, GHB self-administration is represented in yellow, the pilot study and self-administration details are represented in red, the withdrawal period is represented in green, and a temporary cessation of the study due to construction work in the animal facility is represented in black

The methods of this study are reported in compliance with the ARRIVE guidelines (Curtis et al. 2022; Percie du Sert et al. 2020) (Fig. 1).

### Animals

We employed rats in this study because the rat is the preferred species for preclinical addiction research (Homberg, et al. 2017). All rats were pair-housed in enriched type IV cages (59 × 38 × 20 cm; Tecniplast 1500U) with corncob bedding (GM 12 irradiated, Bio Services) under conventional conditions (no filtertops). When GHB was available in the home cage, rats were separated with a perforated plexiglass divider in order to measure individual GHB and water intake. The divider enabled rats to be in closer proximity and in a less stressful environment compared to individual housing. On each side of the divider, one bottle of water and one bottle of GHB were freely available. The rats had ad libitum access to food (dried pellets of standard chow food [ssniff RM V1534-703, Bio Services]) and water. The rats were maintained on a reversed light–dark schedule (lights off at 08:00 h) in temperature- (21 ± 1 °C) and humidity-controlled (55% ± 5%) rooms. The experimental procedures were performed under a project license from the Central Committee on Animal Experiments (Centrale Commissie Dierproeven, The Hague, The Netherlands), in full compliance with the legal requirements of Dutch legislation on the use and protection of laboratory animals (Animal Testing Act). All efforts were made to reduce the number of animals used and their suffering.

### Pilot study

We first conducted a pilot study to (1) determine under which conditions the rats showed maximum voluntary GHB intake, and (2) assess the possible risk for GHB overdosing during home-cage GHB consumption.

#### Animals

In our pilot study, we used 10 Long Evans rats (50% female) (Janvier, France, PND28 on arrival). No power analysis was performed for this pilot study. We based our sample size on GHB consumption seen in Colombo et al. (1995). The weight of the rats was monitored twice-daily during the GHB consumption period.

#### GHB self-administration

All rats had access to GHB in water in their home cage according to a two-bottle choice procedure (GHB and water). On Mondays, the GHB concentration was increased from 1 to 2, 3, 4, and 5% GHB (weight (w)/volume (v)) (Serra et al. 2002). On Mondays and Tuesdays, GHB was available for 6 h per day and rats were monitored for possible GHB overdosing. On Wednesdays, Thursdays, and Fridays, rats had access to GHB for 24 h. GHB and water consumption were measured hourly over a 6-h period by measuring bottle weights. In the weekends, rats only had access to water. The location of the water and GHB bottle was switched every day to prevent side bias.

The concentration that resulted in the highest volume consumed (5% GHB (w/v)) was used in subsequent voluntary GHB intake tests. First, a 5% GHB (w/v) was dissolved in a 5% sucrose solution (w/v) for 1 week to increase palatability of GHB and to examine whether this would increase GHB intake. This sucrose/GHB solution was presented in addition to a regular water bottle.

Hereafter, continuous versus intermittent access of a 5% GHB (w/v) solution (without sucrose) was measured, based on earlier work of Spoelder et al. (2015). First, continuous 5% GHB consumption was assessed for 14 days. Then, intermittent GHB consumption was assessed by providing a GHB bottle for 24 h on Mondays, Wednesdays, and Fridays for 2 weeks. The remaining days, rats only had access to water.

Following the access schedule test (continuous versus intermittent), increasing concentrations of GHB were assessed to examine whether increasing GHB concentrations would automatically yield higher GHB intake. We increased GHB doses from 4% onwards to 5, 6, 7, and 8%, until we observed a lack of a further increase in GHB intake at 8% GHB (w/v). Finally, we examined if the temporary removal of the water bottle (3 h per day) would lead to increased GHB intake (forced GHB), as observed in Colombo et al. (1995).

### Full study

#### Animals

We used 40 Long Evans rats (50% female) (Janvier, France, PND28 on arrival) to establish a GHB self-administration paradigm. As a part of our ethical approval application, we performed an a priori sample size calculation with novel object recognition memory as the main outcome parameter. Based on findings by van Nieuwenhuijzen et al. (2010) (standardized effect size = 1.64), with a power of > 0.80 and an alpha of 0.05, a sample size of 10 rats per group was required. In order to also be able to assess time effects and interactions, and assuming a large effect size of 0.5, a sample size of 18 rats per group was required. Accounting for possible drop-out, we used a total group size of 40 rats. Rats were assigned to either the GHB group or a control group according to a counterbalanced design, based on baseline elevated plus maze (EPM) and novel object recognition (NOR) memory. This lead to similar baseline EPM and NOR scores between treatment groups (male GHB *n* = 10, female GHB *n* = 10, male control *n* = 10, female control *n* = 10). Cage location in the housing room was randomized to prevent unwanted effects caused by the environment. Throughout the study, four rats (one male control, one female control, two female GHB) were taken out of the study due to reaching the humane endpoints (HE) unrelated to GHB consumption (e.g., a broken leg, bacterial infection) (HE1: The animal experiences more discomfort than justified for the purpose of the experiment approved by the local Animal Ethical Committee (unrelievable adverse behavioral changes, unrelievable adverse body condition, weight loss > 20%); HE2: The animal experiences more than little, additional, discomfort as a result of conditions not related to the experiment). The exact number of rats that was used in each experiment is provided in the respective figure legends.

#### Home-cage GHB consumption

Rats in the GHB group had continuous access to 7% (w/v) GHB (Xyrem 500 mg/mL, Jazz Pharmaceuticals, Athlone, Ireland) in water according to a two-bottle choice procedure. GHB and water were presented in drinking bottles with stainless steel dual ball-bearing drinking spouts for 3 months. These GHB access parameters were determined in the pilot study. The location of the GHB bottle and water bottle was switched from left to right (or vice versa) once per week to prevent side bias. The drinking bottles were weighed daily on Mondays until Fridays and the body weight of the rats was measured once a week.

#### Behavioral assessment of GHB intoxication and withdrawal

To assess potential GHB intoxication, individual behavioral parameters (locomotion, immobility, grooming, rearing, drinking, and eating) and the aberrant behaviors wet dog shakes, head shakes, and head twitching were automatically classified and assessed during GHB access in regular Type-IIIH cages using LABORAS (Metris B.V., Hoofddorp, The Netherlands); Laboratory Animal Behavior Observation Registration and Analysis System (Castagné, et al. 2012). The intoxication measurements were conducted during two 3-h sessions, performed after 1 and 2 months of home-cage GHB access. Throughout these sessions, the rats had free access to food. The GHB group had access to GHB, while the control group had access to water. The measurements were performed during the rats’ active phase.

To assess withdrawal, GHB was removed from the home cage directly following the 3-month GHB consumption period, and behavior was assessed using LABORAS for 22 h.

#### Operant GHB self-administration

Rats that previously had access to GHB in the home cage underwent behavioral training and testing in operant boxes (29.5 cm L, 24 cm W 25 cm H; Med Associates, Georgia, VT), situated in light- and sound-attenuating cubicles equipped with a ventilation fan. Each box was equipped with an active and an inactive 4.8-cm-wide retractable lever, placed 11.7 cm apart and 6 cm from the grid floor. The location of the active and inactive lever was counterbalanced between rats. A cue light was present above each lever and a liquid dipper was located between the levers. Upon an active lever press, the dipper cup containing GHB (0.1 mL, 7% w/v) was raised for 10 s. Simultaneously, a cue light lit up for 15 s, during which additional presses on the active lever had no consequences. Pressing the inactive lever was recorded, but had no programmed consequences. Head entries in the reward receptacle were measured through infrared beam breaks.

**Fig. 2:**
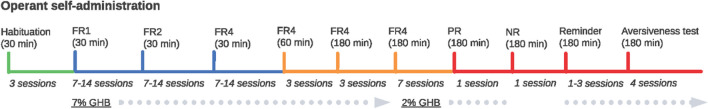
Schematic representation of the operant self-administration experiments

Rats were habituated to the operant box for three daily 30-min habituation sessions, during which no levers or cues were present. Rats were subsequently trained to press the lever according to fixed ratio 1 (FR1), FR2, and FR4 schedules of reinforcement (seven to 14 sessions per schedule) (Fig. 2). The response requirement was increased after a minimum of seven sessions, and when rats earned at least one reward in three subsequent sessions. To increase behavioral output, the duration of the FR4 operant sessions was increased from 30 min (seven to 14 sessions) to 1 h (three sessions) and 3 h (three sessions with 7% GHB (w/v)). Seven additional 3-h FR4 sessions were conducted using a 2% GHB (w/v) concentration to examine whether rats would increase their lever active presses, seeking comparable GHB effects as observed with the 7% GHB concentration (Fig. 2). The rats were tested 5 days per week, once per day. Rats that did not earn a reward in three subsequent sessions (*n* = 6) were excluded from further training and testing.

#### Assessment of motivation to use, habitual drug-seeking and use despite negative consequences

The motivation to use GHB was tested on a 3-h progressive ratio (PR) schedule of reinforcement, during which the number of required active lever presses was calculated according to the formula $F \left(n\right)=5 \times {\text{EXP}}\left(0.2n\right)-5$. Following PR, a 3-h non-reinforced session (NR) was performed, in which an active lever press had no programmed consequences. After the NR session, one to three 30-min FR4 reminder sessions were performed to reinstate GHB-seeking behavior. When a rat earned at least one GHB presentation in a reminder session, we examined GHB use despite negative consequences. We adulterated GHB with increasing doses of quinine, from 0.0 to 0.1, 0.3, and 1.0 g/L quinine in 2% GHB (w/v). Each 3-h quinine session was performed under a FR4 schedule of reinforcement.

#### Elevated plus maze

Anxiety-like behavior was measured before and directly after home-cage GHB consumption using the elevated plus maze (EPM) (Fig. 1) (Walf & Frye, 2007). The maze, elevated 50 cm from the floor, consisted of two open arms (50 × 10 cm, 10 lx) and two closed arms (50 × 10 cm) that were enclosed by a side wall. Rats were placed in the center of the maze, facing the open arm and were allowed to explore the apparatus for 5 min, while being recorded by a camera suspended above the center of the maze.

#### Novel object recognition

Object recognition was tested before, and 13 weeks after home-cage GHB consumption using the novel object recognition (NOR) test. The second assessment of the NOR (after GHB exposure) was preceded by a 10-week reconstruction period at the animal facility (Fig. 1). Habituation, training, and testing were performed according to Genzel et al. (2019). In short, rats were habituated to a white MDF testing box (80 × 80 × 80 cm) during five daily 10-min sessions. During the first three sessions, no cues or objects were present in the testing box. In sessions four and five, two objects (made from Duplo blocks, not used in main experiment) were placed in the testing box and rats were allowed to explore. On the bottom side of the floor of the testing box, four magnets were placed in fixed locations for consistent placement of the objects. The objects were attached to square metal plates. During training, two identical objects were placed in the testing box and rats were allowed to explore the testing box for 10 min. The object type during training and the location of the objects were counterbalanced between rats. Following an inter-trial interval of 24 h, one of the objects was replaced with another object, after which the rats were again allowed to explore the testing box for 10 min. Rats were recorded using an overhead video camera system.

#### Data and statistical analysis

The data and statistical analyses complied with the recommendations on experimental design and analysis in pharmacology (Curtis et al. 2022). All rats tested were treated as independent values, there were no technical replicates.

Performance on the NOR task was determined by calculating the Discrimination Index (DI). The DI was calculated as the difference between the time exploring the novel object and the old object, divided by the total exploration time. Videos were analyzed using the automated tracking software DeepLabCut combined with a custom-written Python script, providing unbiased data analysis (Mathis et al. 2018). Exploration time was determined as the total duration that the nose of the rat was within eight pixels of the object. When the center of the body was in the object area (i.e., rat was sitting on the object), it was not counted as exploration time.

For the EPM, total time spent in the center, open and closed arms, and latency to enter the open arms were automatically quantified using Observer Ethovision (Noldus, Wageningen, The Netherlands).

The effect of baseline anxiety-like behavior (EPM) on home-cage GHB consumption was analyzed with a linear regression model, using the AUC for the 3-month home-cage GHB consumption as the dependent variable and the relative time spent in the closed arms as the predictor variable.

Typical withdrawal symptoms (wet dog shakes, head shakes, heat twitches, and scratching) during the first 3 h of abstinence in GHB rats were compared with a 3-h LABORAS session of control rats (performed 2 months after home-cage self-administration).

Performance on operant sessions was represented as active lever presses, GHB presentations (number of successful completions of the schedule of reinforcement), and GHB consumptions (head entries directly following a GHB presentation). An addiction severity score was computed based on home-cage GHB intake (area under the curve (AUC) for GHB intake (mg/kg)), habitual drug-seeking (active lever presses under non-reinforced conditions divided by active lever presses under reinforced conditions), motivation to use (active lever presses during PR), and use despite negative consequences (AUC for GHB intake (mg/kg) under increasing concentrations of quinine) (Belin et al. 2009). The four measures were normalized by subtracting the mean of all rats from the score of every individual rat. Thereafter, we divided this ratio by the standard deviation of the whole group. The sum of three out of four measures was calculated for every combination. For every combination, the highest quartile (*n* = 3) was identified as exhibiting addiction-like behavior. For a more detailed explanation, see Smeets et al. (2022).

Where appropriate, each parameter was tested for normality with a Shapiro–Wilk test. Mauchly’s test of sphericity was used to test whether variances of the differences between treatment levels were equal. If the assumption of sphericity was violated, or when dealing with repeated measures, a Geisser-Greenhouse correction was applied. Normally distributed behavioral data were analyzed via two-sample *t*-tests, one-, two-, or three-way ANOVAs with time or session as within-subject factor, and drug (and sex where appropriate) as between-subject factors, unless indicated otherwise. Correlation analyses were performed between home-cage GHB consumption, motivation to use GHB, habitual drug-seeking and use despite negative consequences, resulting in six individual correlation analyses. Bonferroni correction was performed to correct for multiple comparisons (*p* = 0.05/6 = 0.0083). For correlation analyses involving motivation to use GHB, one rat was identified as an outlier and was excluded from the analyses (deviating > 2 × SD from the group mean). Bonferroni post hoc analyses were also performed following planned comparisons or following significant ANOVA interactions, and were only performed if data were normally distributed and if there was no inhomogeneity of variance. Experimenters were blinded during manual data analysis.

The threshold for statistical significance was set at *p* < 0.05. All data are presented as mean ± SEM. Statistical analyses were conducted using GraphPad Prism (version 10.0).

## Results

### Pilot study

We did not witness GHB overdosing following home-cage GHB consumption. Maximum average GHB intake was established at 7% GHB in a water (w/v) solution under continuous free-choice access, without addition of sucrose (Suppl. Figure 1). These access parameters were employed in the full study.

### Full study

#### Home-cage GHB consumption

**Fig. 3:**
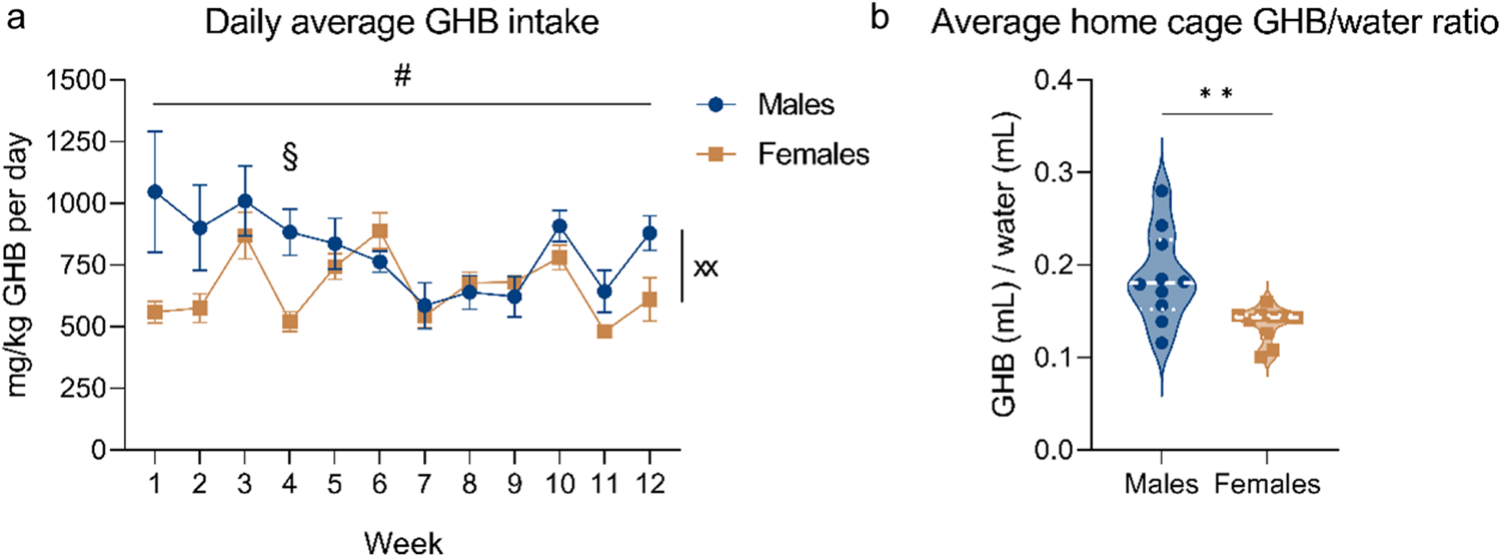
Home-cage GHB consumption over a period of 12 weeks. **a** mg/kg GHB intake during the self-administration period of 12 weeks, divided by sex. **b** GHB preference normalized by water intake for males and females, averaged over the entire self-administration period. Males *n* = 10, females *n* = 10. # = *p* < 0.05 two-way RM ANOVA main effect of time; xx = *p* < 0.01 time × sex interaction; § = *p* < 0.05 Bonferroni multiple comparison test; ** = *p* < 0.01 two-sample *t*-test

Rats showed pharmacologically relevant (> ~ 87.5 mg/kg per acute dose (Martellotta et al. 1997)) levels of daily GHB intake during home-cage consumption (males: *μ* = 809.7 mg/kg, SD = 226.7; females: *μ* = 660.6 mg/kg, SD = 92.0), although the dose for each consumption is difficult to accurately estimate. GHB intake fluctuated over a period of 12 weeks, and the GHB intake of males was higher compared to females during week 4 (Fig. 3a: two-way ANOVA, time × sex interaction, *F*_(11, 198)_ = 2.769, *p* < 0.01, Bonferroni post hoc test week 4, *p* < 0.05). No difference in overall GHB intake was observed between males and females over a period of 12 weeks. Male rats exhibited a higher preference for GHB versus water compared to females (Fig. 3b: two-sample *t*-test, *p* < 0.01).

#### Behavioral assessment of GHB intoxication and withdrawal

##### Intoxication

No clear intoxication symptoms were visible during the 3-h sessions of GHB consumption in LABORAS (Suppl. Figure 2, three-way ANOVA, no effect of drug, *p* > 0.05). No sex-specific drug effects were observed (Suppl. Figure 2, three-way ANOVA, no sex × drug interaction, *p* > 0.05). However, we did see a differential effect of GHB on home-cage behaviors (Suppl. Figure 2, three-way ANOVA, behavior × drug interaction, *F*_(5, 216)_ = 6.567, *p* < 0.0001). Therefore, we compared GHB vs control for every behavior with males and females grouped. GHB rats spent less time eating compared to controls (Suppl. Figure 2, Bonferroni post hoc comparison, GHB vs control eating, *p* < 0.05).

**Fig. 4:**
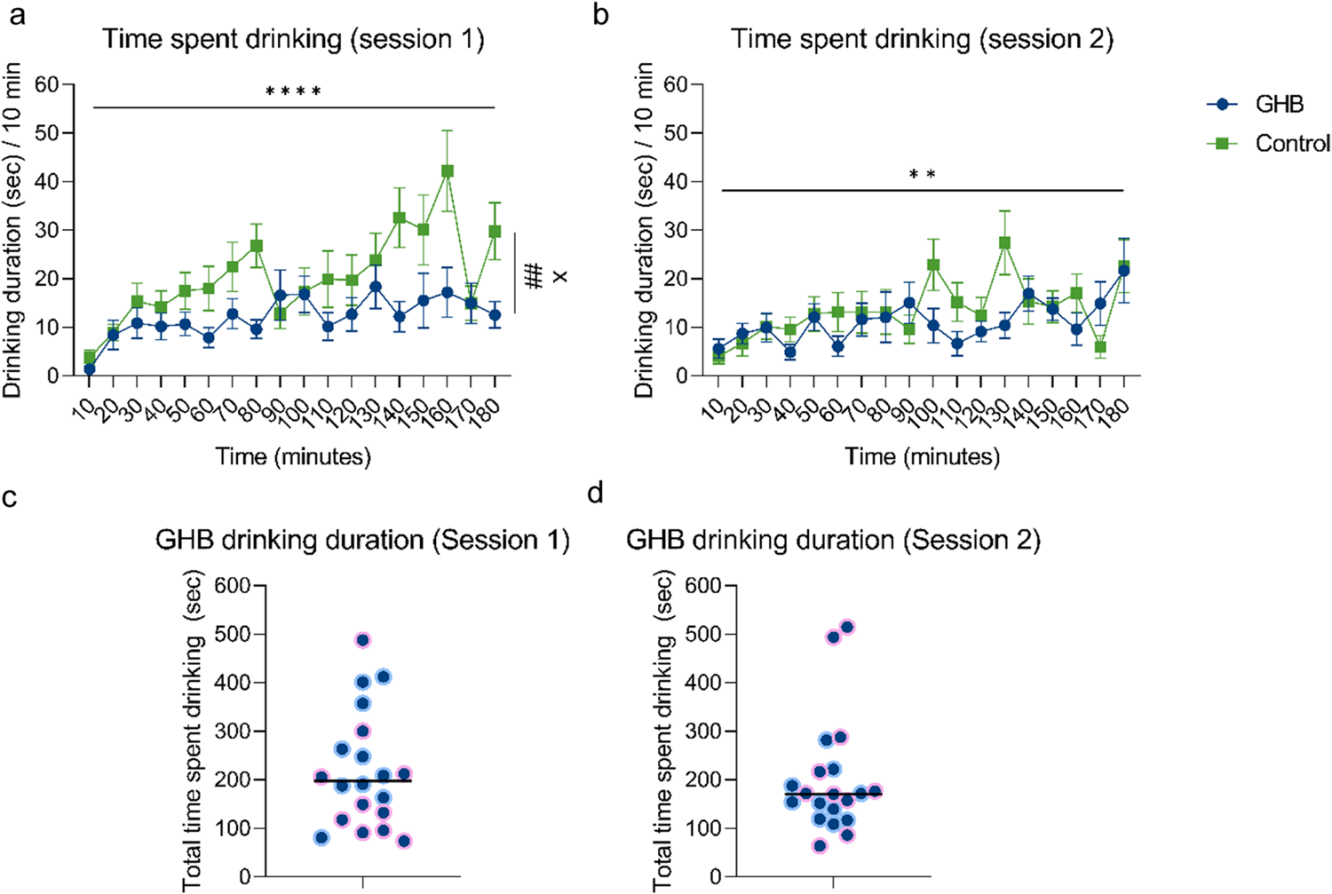
**a** Time spent drinking during 3-h LABORAS sessions after 1 month (session 1) in 10-min bins. **b** Time spent drinking during 3-h LABORAS sessions after 2 months (session 2) in 10-min bins. GHB rats only had access to GHB, while control rats only had access to water. **c** Total time spent drinking GHB for each individual GHB rat during session 1. **d** Total time spent drinking GHB for each individual GHB rat during session 2. Blue dots represent male rats, and pink dots represent female rats. GHB *n* = 20, control *n* = 20. ** = *p* < 0.01, **** = *p* < 0.0001 two-way RM ANOVA main effect of time; ## = *p* < 0.01 main effect of drug, x = p < 0.05 time × drug interaction

Rats showed stable consumption of GHB during 3-h GHB sessions, both after 1 month of home-cage GHB consumption (session 1, Fig. 4a) and 2 months of home-cage GHB consumption (session 2, Fig. 4b). During session 1, water consumption in the control group exceeded GHB consumption in the GHB group, which did not have access to water during the LABORAS sessions (Fig. 4a, two-way ANOVA, main effect of group, *F*_(1, 38)_ = 9.799, *p* < 0.01). Time spent drinking GHB varied from ~ 50 s up to ~ 500 s between rats during a 3-h period (Fig. 4c session 1, *μ* = 219.0 s, SD = 119.4; Fig. 4d session 2, *μ* = 199.7 s, SD = 118.5). Additionally, some rats showed binge-like periods of GHB consumption, i.e., distinct peaks in GHB consumption (Suppl. Figure 3).

##### Withdrawal

**Fig. 5:**
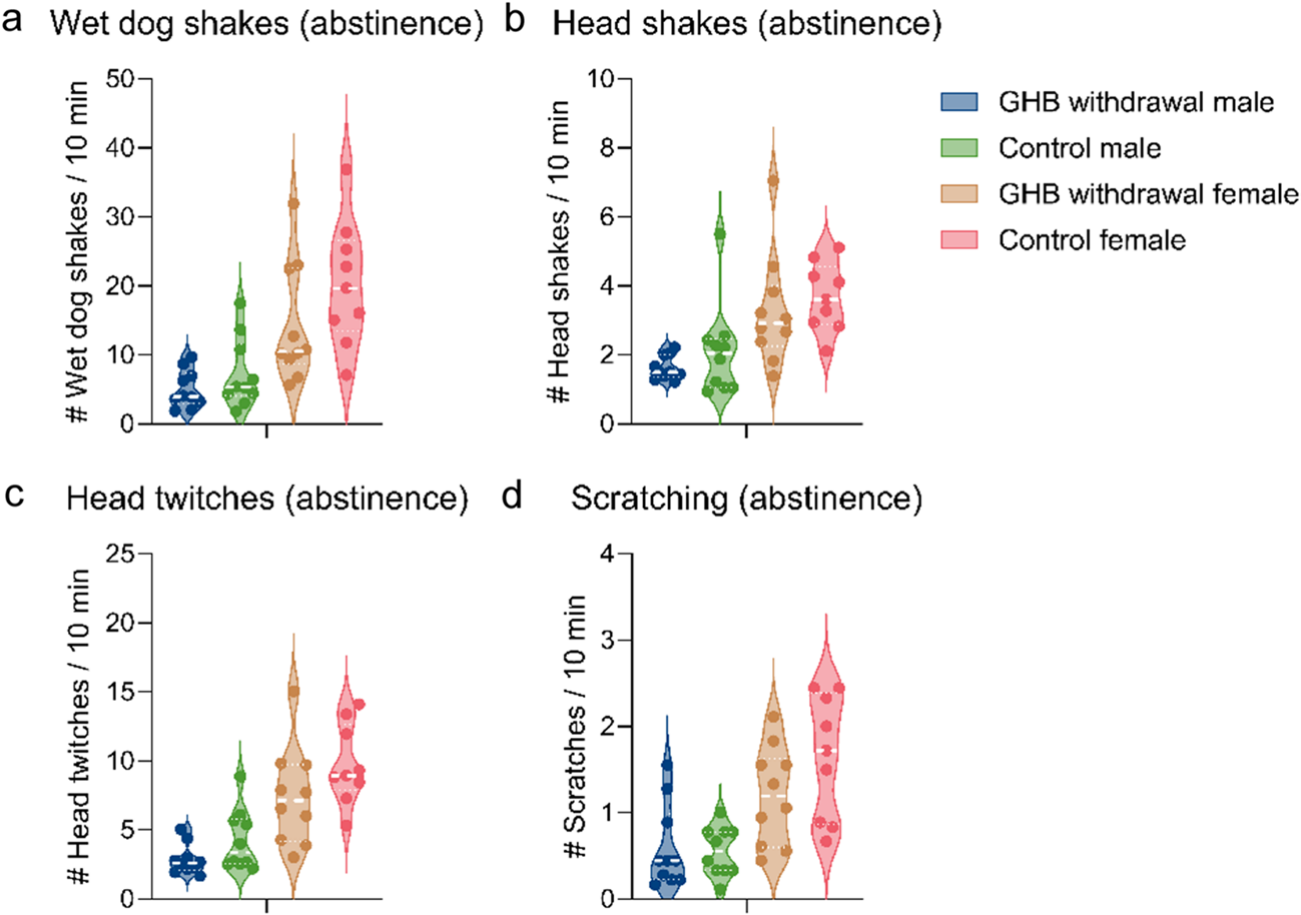
Aberrant behaviors during 3 h of sudden abstinence of the GHB group, compared to a 3-h session of control rats. **a** Number of wet dog shakes averaged per 10 min. **b** Number of head twitches averaged per 10 min. **c** Number of head shakes averaged per 10 min. **d** Number of scratches averaged per 10 min

We did not observe a difference in the number of wet dog shakes, head shakes, heat twitches, or scratches during the first 3 h of GHB abstinence compared to a control group (Fig. 5, Bonferroni post hoc comparisons, male GHB vs male control, *p* > 0.05; female GHB vs female control, *p* > 0.05). Upon visual inspection of the 22-h development of locomotion, rearing, grooming, wet dog shakes, head shakes, and head twitches following abstinence, no clear indications of withdrawal were observed (Suppl. Figure 4). Both males and females showed a decrease in all behaviors during the dark phase, indicating a regular day-night rhythm unaffected by abstinence. Females showed higher behavioral output compared to males during withdrawal, as similar during intoxication (Suppl. Figure 2).

#### Operant GHB self-administration

**Fig. 6:**
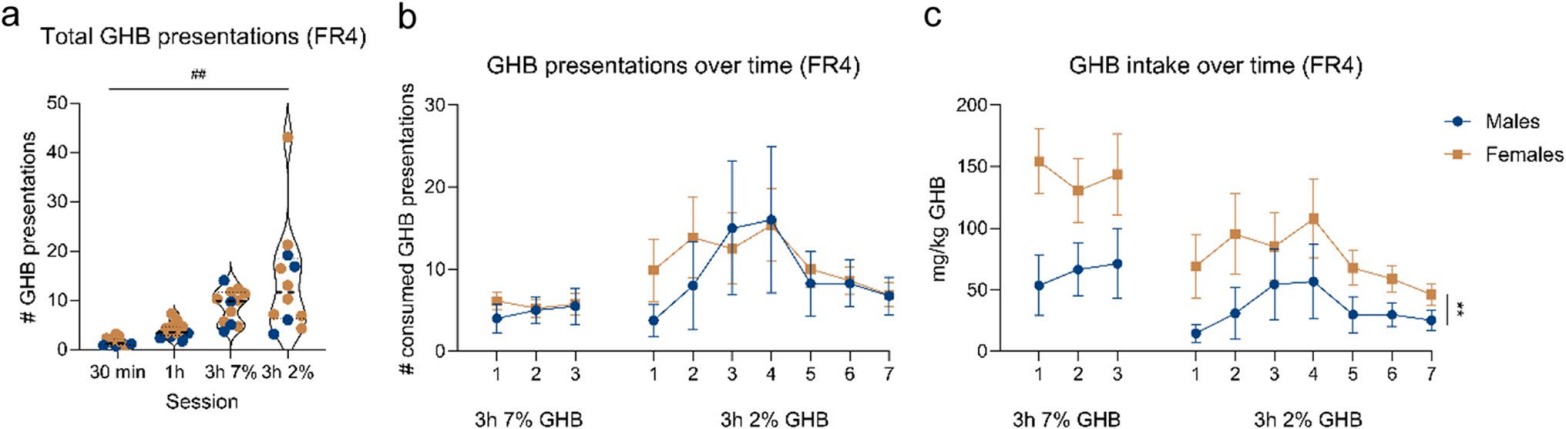
GHB presentations and intake during 3-h FR4 sessions. **a** Total GHB presentations during different FR4 sessions. Blue data points correspond to males, and pink data points correspond to females. **b** Consumed GHB presentations per 3-h session, divided by sex. **c** mg/kg GHB intake per 3-h session, divided by sex. Males *n* = 4, females *n* = 8. ## = *p* < 0.01 one-way ANOVA main effect. ** = *p* < 0.01 Welch’s two-sample *t*-test males vs females

The number of GHB presentations increased when the operant session duration increased (Fig. 6a, one-way ANOVA, main effect of session, *F*_(1.158, 12.73)_ = 13.36, *p* < 0.01). Males and females showed a similar number of (consumed) GHB presentations (Fig. 6b). Due to a lower bodyweight of the female rats, this resulted in a higher mg/kg GHB consumption for females compared to males (Fig. 6c, Welch’s two-sample *t*-test, males vs females, *p* < 0.01). Throughout the operant FR4 sessions, active lever presses were higher than inactive lever presses (Suppl. Figure 5: two-way ANOVA, main effect of lever type, *F*_(2, 35)_ = 11.04, *p* < 0.001). After switching from 7 to 2% GHB, an increase and subsequent decrease in GHB presentations and GHB intake over sessions were observed (Fig. 6b, Fig. 6c, Suppl. Figure 5). The number of GHB presentations and GHB intake during 3-h 7% GHB sessions remained stable (Fig. 6b, Fig. 6c).

**Fig. 7:**
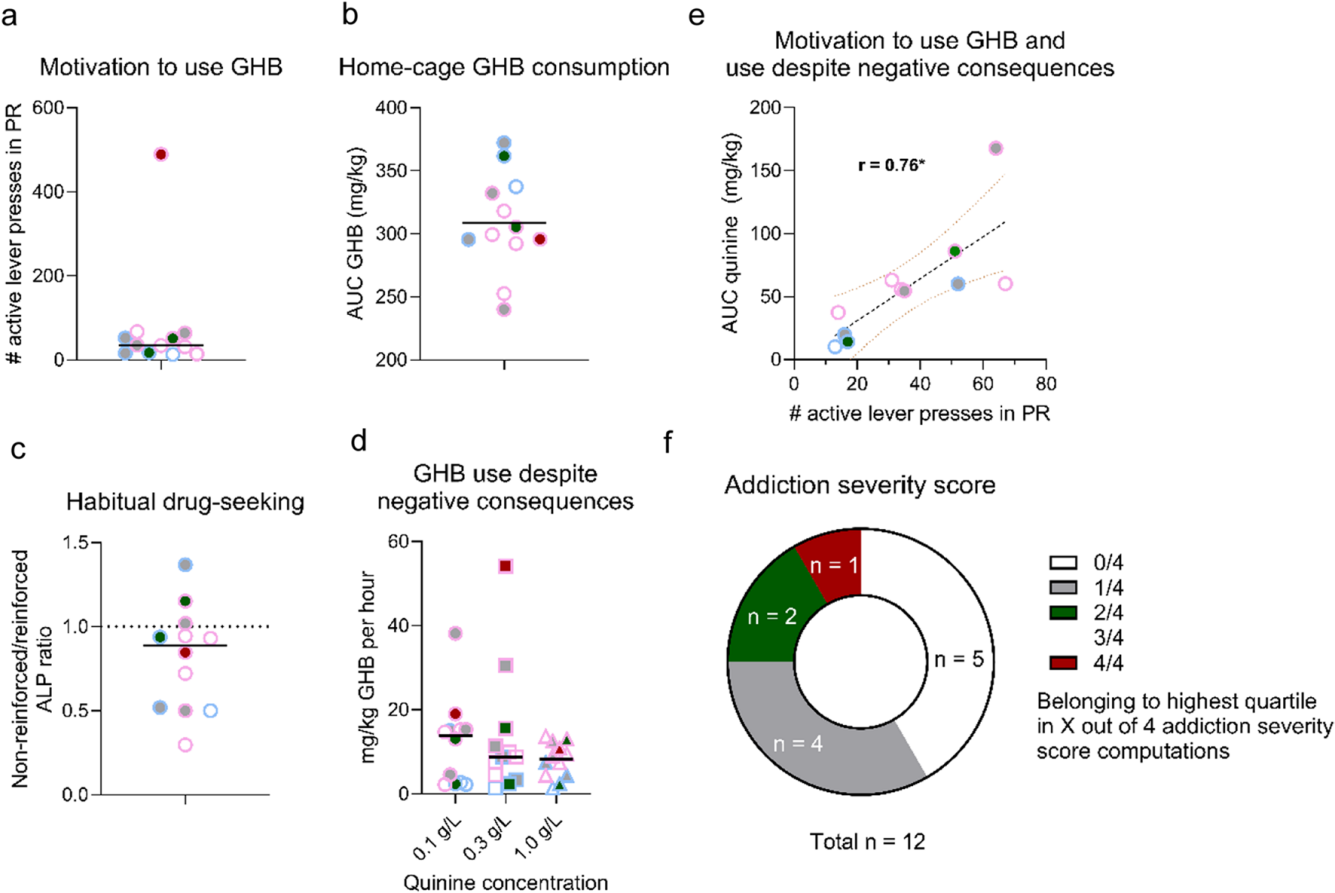
Addiction-like behaviors for individual rats. **a** Area-under-curve (AUC) for 3-month home-cage GHB intake. **b** Motivation to use GHB, represented by the active lever presses during the 3-h progressive ratio (PR) schedule of reinforcement test. **c** Habitual drug-seeking, calculated by the active lever presses during the non-reinforced session compared to the amount of active lever presses (ALP) during previous reinforced FR4 schedule of reinforcement. **d** GHB use despite negative consequences, represented by GHB consumption during 3-h operant sessions with different doses of quinine-adulterated GHB. **e** Correlation between motivation to use GHB and use despite negative consequences (*n* = 11). * = *p* < 0.0083, Bonferroni correction. Black line represents the regression line, and curved dotted lines represent the 95% confidence interval. **f** Distribution of addiction severity scores, represented by the frequency of belonging to the highest quartile in the 4 addiction severity score computations. Data points represent individual animals. Symbol border color corresponds to the sex of the individual rat. Addiction severity score colors are used as symbol colors in **a**–**e**

Rats that finished operant training (*n* = 12) were tested for several addiction-like behaviors. During PR testing, one rat showed extremely high responding (489 active lever presses during a 3-h session) (Fig. 7a). Active lever responses of the majority of rats during the PR schedule of reinforcement were comparable to active lever responses during FR4 (varying between 13 and 67) (Fig. 7b). Responding under non-reinforced conditions was slightly decreased compared to reinforced responding, although responding was still maintained (average ratio of 0.81) (Fig. 7c). Quinine-adulterated GHB decreased operant responding on the active lever in a dose-dependent manner. Two rats still showed responding with 0.1 g/L and 0.3 g/L quinine in GHB, whereas responding for GHB completely diminished in all rats with 1.0 g/L quinine (Fig. 7d). Motivation to use GHB strongly correlated with use despite negative consequences (*r* = 0.76, *p* < 0.0083) (Fig. 7e). Other addiction-like behaviors were not correlated with each other (Suppl. Figure 6).

To assess and quantify the presence and consistency of addiction-like behavior, we calculated the addiction severity score for every individual rat. One rat was represented in the highest quartile of all addiction severity score computations (one female), two rats were represented in the highest quartile of two addiction severity score computations (one male, one female), while four rats were represented in the highest quartile in one of the four of the addiction severity score computations (two males, two females). Forty-two percent of the tested rats (5/12) were not represented in the highest quartile of any of the addiction severity score computations (one male, four females) (Fig. 7f).

#### Object recognition memory and anxiety-like behavior

**Fig. 8:**
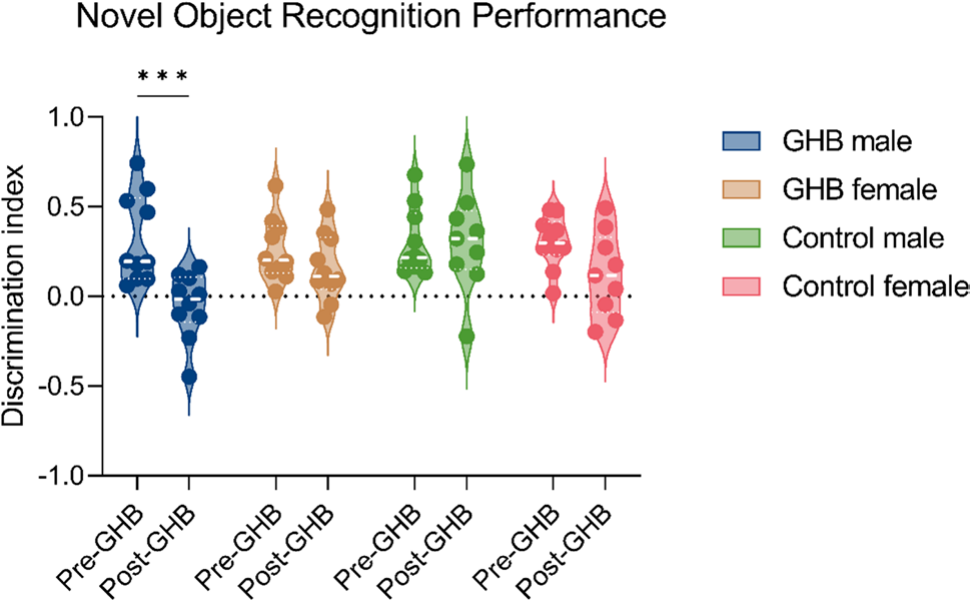
Novel object recognition (NOR) performance before and after a 3-month home-cage GHB self-administration period. Baseline (pre-GHB) NOR was tested in the 2 weeks prior to home-cage GHB access. Post-GHB NOR performance was tested under abstinence, 13 weeks after home-cage GHB self-administration. GHB male *n* = 10, GHB female *n* = 10, control male *n* = 10, control female *n* = 10 (*n* = 9 for female control post-GHB). *** = *p* < 0.001 Bonferroni multiple comparison

We explored the effect of home-cage GHB intake on object recognition memory. Baseline object recognition memory was similar between the GHB group and the control group (Fig. 8, three-way ANOVA, no effect of group *p* > 0.05). Three-month home-cage GHB intake led to decreased object recognition memory after 13 weeks of abstinence in males, whereas object recognition performance was unaffected in females (Fig. 8: three-way ANOVA, group × sex × intervention interaction, *F*_(1, 34)_ = 7.621, *p* < 0.01, Bonferroni post hoc comparison, GHB male pre-GHB vs post-GHB, *p* < 0.001). Home-cage GHB intake was not predictive of novel object recognition performance (Suppl. Figure 7, linear regression, *p* > 0.05). We also explored the effect of baseline anxiety-like behavior on home-cage GHB intake and vice versa. We did not observe a predictive effect of baseline anxiety-like behavior on home-cage GHB intake (Suppl. Figure 8a). Home-cage GHB consumption and subsequent abstinence did not affect anxiety-like behavior (Suppl. Figure 8b, c, d, e).

## Discussion

This study set out to assess the characteristics of oral GHB self-administration in outbred rats. GHB self-administration was primarily characterized by strong inter-individual differences between rats. All rats showed intake of pharmacologically relevant levels (> 87.5 mg/kg) of GHB in a home-cage environment. We did not observe escalation in GHB intake, physical intoxication symptoms, or withdrawal symptoms after sudden GHB abstinence. A subset of rats showed motivation for GHB or GHB use despite negative consequences, while over half of the rats showed habitual GHB-seeking in an operant environment. Chronic home-cage GHB intake led to a decrease in long-term memory performance in male rats. Baseline anxiety-like behavior was not predictive of home-cage GHB intake, while GHB did not affect anxiety-like behavior following abstinence.

Over a period of 12 weeks of home-cage GHB access, rats showed stable consumption of pharmacologically relevant amounts of 7% GHB (w/v) (~ 700 mg/kg/day) with an average GHB preference of ~ 15–20%. This stable GHB intake over time is in line with the majority of human GHB users that do not show escalation (Dijkstra et al. 2021). In addition, patients who daily consume GHB for medical purposes (e.g., narcolepsy patients) also reportedly do not show signs of tolerance or dose escalation (Wang et al.  2009). Our findings are also largely in line with preclinical GHB literature. A study by Colombo et al. (1995) found no increase in 1% GHB (w/v) consumption during 20 days of free-choice GHB access in outbred Wistar rats (Colombo et al. 1995). In selectively bred ethanol-preferring male rats, 1% GHB (w/v) intake did increase over a period of 28 days (from ~ 200 mg/kg/day up to ~ 600 mg/kg/day), but GHB intake in non-ethanol-preferring male rats remained stable over time (~ 200 mg/kg/day) (Colombo et al. 1998). GHB preference varied from ~ 20% in non-ethanol-preferring rats (as observed in our study) to ~ 50% in ethanol-preferring rats at the end of the 28-day period. Altogether, this indicates that escalation in GHB use may only occur in a subset of rats that are susceptible for the development of addiction-like behavior, similar to the human situation. Future studies should examine the use of selectively-bred animals to assess GHB escalation and tolerance in a self-administration model.

Besides decreased food consumption, no other GHB intoxication symptoms were detected after 1 or 2 months of GHB self-administration. Rats showed clear individual differences in total GHB consumption and GHB consumption pattern (binge-like drinking vs stable consumption) during 3-h LABORAS sessions after 1 and 2 months of home cage GHB consumption. One other preclinical GHB study characterized the (hourly) drinking pattern of GHB, reporting binge-like behavior in some rats on some days (Colombo et al. 1995). Unfortunately, the study does not report whether these binges are representative for the entire sample. This binge-like drinking behavior is also observed in patients with GUD, in order to prevent withdrawal symptoms (Beurmanjer et al. 2019). Although we see binge-like consumption in some rats, the majority of rats in our sample exhibit controlled, stable GHB consumption. This may resemble the recreational GHB consumption pattern that is seen in the broader population of GHB users, unrelated to the occurrence and prevention of withdrawal (Dijkstra et al. 2021).

GHB rats did not show clear withdrawal symptoms when comparing the first 3 h of abstinence with a control group. No aberrant behaviors across a 22-h period of abstinence were observed. In rats, it has been shown that abstinence following 10 repeated injections of 500 mg/kg GHB induces anxiety-like behavior in male rats (van Nieuwenhuijzen et al. 2010), which is also seen in patients with GUD undergoing controlled detoxification (Wolf et al. 2021). Additionally, 24-h abstinence following 18 twice-daily doses of 1.5–3.5 g/kg GHB in selectively bred alcohol-preferring male rats was shown to induce audiogenic seizures in 60% of the rats (Carai et al. 2005). Seizures are a hallmark of sudden GHB withdrawal in patients with GUD consuming high doses of GHB (McDonough et al. 2004) and are also observed following withdrawal from other sedatives like alcohol and benzodiazepines (Rogawski 2005). However, when rats were tested for different types of withdrawal symptoms (including hyperlocomotion, anxiety-like behavior, tremors, and head shakes), we observed no clear signs of GHB withdrawal. Despite differences in administration procedures and the specific parameters that were assessed, our results correspond to the lack of withdrawal symptoms in recreational GHB users in contrast to patients with GUD. This is in line with the suggestion that our model does not resemble the phenotype of GUD, but may resemble recreational, habitual GHB use instead.

We were able to establish consistent and stable operant responding for GHB in two-thirds of the rats (*n* = 12) that were exposed to GHB in an operant environment. Motivation to use GHB and use despite negative consequences were strongly correlated in our model, indicating internal consistency in (operant) GHB addiction-like behavior. Only one rat (~ 10%) showed consistent addiction-like behavior across all four tests. Smeets et al. (2022) employed a similar approach to assess several addiction-like behaviors in an alcohol self-administration model. They did not find a correlation between motivation to use and use despite negative consequences. However, despite differences in experimental design, they found that ~ 10% of the rats showed consistent addiction-like behavior. Similar to our study, the majority of their rats did not exhibit any addiction-like behaviors despite the availability of a reinforcing drug. Other pre-clinical studies also found clear differences in addiction-like behavior between outbred rats with access to reinforcing drugs (e.g., high vs low responders, high-drinking animals vs low-drinking animals) (Bell et al. 2006; Kabbaj and Targets 2006; Spoelder et al. 2015). This variance in addiction-like behavior is also observed in the general population, where it is known that a small subset of people that recreationally use, e.g., alcohol develop a substance use disorder (~ 15%) (Grant et al. 2017). Specifically, for GHB users, three subpopulations are described in the literature: people using GHB recreationally without adverse effects (most prevalent), people using GHB recreationally with adverse effects (less prevalent), and people with dependence on GHB (least prevalent) (Dijkstra et al. 2021). These subgroups may be represented in our model, with the majority of rats consuming GHB without measurable adverse effects. Future studies should further develop our model to allow the study of more prominent addiction-like behavior. Additionally, future studies may also explore the subset of rats that do not show any signs of addiction-like behavior, despite the availability of a drug with abuse potential. This may provide us novel insights regarding the resilience in development of GUD and substance use disorder in general.

Despite the limited presence of GUD-like characteristics, 3-month home-cage GHB access led to a sex-specific decrease in novel object memory compared to a control group. Memory performance following a 24-h interval was tested 13 weeks after the final exposure to GHB, indicating a long-lasting residual effect on long-term memory performance (Akkerman et al. 2014). In line with our findings, another preclinical study found that ten daily injections of 500 mg/kg GHB in rats (similar to the daily intake observed in our study) caused decreased spatial long-term memory performance 5 days after the final injection (Chen et al. 2017). In patients with GUD, long-term memory performance has not yet been studied, in contrast to other memory domains. GUD patients showed impairment in verbal short-term memory performance, unrelated to severity of GUD or number of comas (Beurmanjer et al. 2022). Another study could not find this association between GHB use and verbal short-term memory impairment, or an association between GHB use and spatial short-term memory impairment (Pereira et al. 2018). However, in this study, GHB-induced comas were associated with decreased verbal short-term memory (Pereira et al. 2018). Altogether, it appears that long-lasting and possibly irreversible cognitive effects can arise following chronic GHB administration. It remains unknown through what mechanisms these cognitive effects occur. The current model provides the means to study these mechanisms in a controlled setting, and allow for the exploration of potential preventive measures. Future studies should examine the underlying mechanisms of the residual negative effects on long-term memory performance, and further study potential negative cognitive effects of chronic GHB use in a patient population.

The current findings should be viewed in light of several study limitations. Responding during the operant sessions (under FR, PR, and NR) was relatively low compared to other addictive substances, such as alcohol (Smeets et al. 2022; Spoelder et al. 2015). This may give rise to the impression that GHB may be less reinforcing than alcohol. However, an important factor to take into account is that here, we established one of the first oral GHB self-administration paradigms in outbred rats, while operant alcohol self-administration has been studied and optimized for decades. It has been suggested that especially the use of selectively bred alcohol-preferring rats is of high value in studying AUD (Borruto et al. 2021), indicating that the use of GHB-preferring rats can be of value in future GHB-related experiments (Lobina et al. 2004).

Since our control rats were not placed in LABORAS following the 3-month self-administration period, we compared symptoms during the first 3 h of abstinence/withdrawal to a 3-h LABORAS session in control rats performed 1 month earlier. Due to the lack of 22-h data from the control group during abstinence, it is possible that we overlooked the presence of withdrawal symptoms. Our interpretation of the results may therefore be an underestimation of GHB withdrawal in our paradigm.

Finally, we quantified operant GHB consumption by registering the number of obtained GHB rewards, and verified whether they were followed-up with a head entry to collect the GHB reward. We opted for this output parameter instead of measuring GHB in the GHB holding container, since evaporation of GHB strongly contributed to the amount of GHB removed from the holding container. Using the number of rewards and the timing of head entries to calculate the amount of consumed GHB might lead to a slight overestimation of the actual amount of consumed GHB. However, in earlier work of operant (alcohol) self-administration, Spoelder et al. (2015) have shown that the number of rewards strongly correlates with the actual volume of consumed 20% alcohol solution (w/v). Based on these results, we expect that the number of rewards and the actual GHB that rats consumed would also correlate. Therefore, we believe that the impact of the possible overestimation of consumed GHB on our results is minimal.

## Conclusion

Oral voluntary GHB self-administration in outbred rats leads to habitual, controlled consumption characterized by inter-individual differences, which is also observed with other addictive substances such as alcohol. In contrast to human GHB users, rats consuming GHB did not show overdosing and subsequent GHB-induced comas. Despite the chronic intake of pharmacologically relevant levels of GHB, no clear intoxication or withdrawal symptoms were observed. Only few rats showed consistent addiction-like characteristics. Our model may thus represent a genetically diverse population sample where the majority does not develop addiction-like behavior. This is further supported by the absence of a relation between anxiety and GHB consumption. Chronic GHB exposure led to residual long-term memory effects in rats, calling for further research into the potential negative cognitive effects of chronic GHB use in humans. This voluntary GHB self-administration model in rats allows for the study of the mechanisms involved in the development of addiction-like GHB use, and enables the exploration of potential preventive strategies.

### Supplementary Information

Below is the link to the electronic supplementary material.

Supplementary file1 (DOCX 1592 KB)

## Data Availability

The data that support the findings of this study are available from the corresponding author upon reasonable request. Some data may not be made available because of privacy or ethical restrictions.

## Declarations

### Disclaimer

The funders had no role in the design of the study; in the collection, analyses, or interpretation of data; in the writing of the manuscript; or in the decision to publish the results.
