# Supplementary material for: Individual differences in GHB consumption in a new voluntary GHB self-administration model in outbred rats
Source: Psychopharmacology (Berl). 2024 Feb 9;241(3):613–25. doi: 10.1007/s00213-024-06537-5 (PMC10884067; doi:10.1007/s00213-024-06537-5)
Supplement: Supplementary file 1 — Supplementary file1 (DOCX 1592 KB) [file 213_2024_6537_MOESM1_ESM.docx]

**Supplementary figures**

a

b


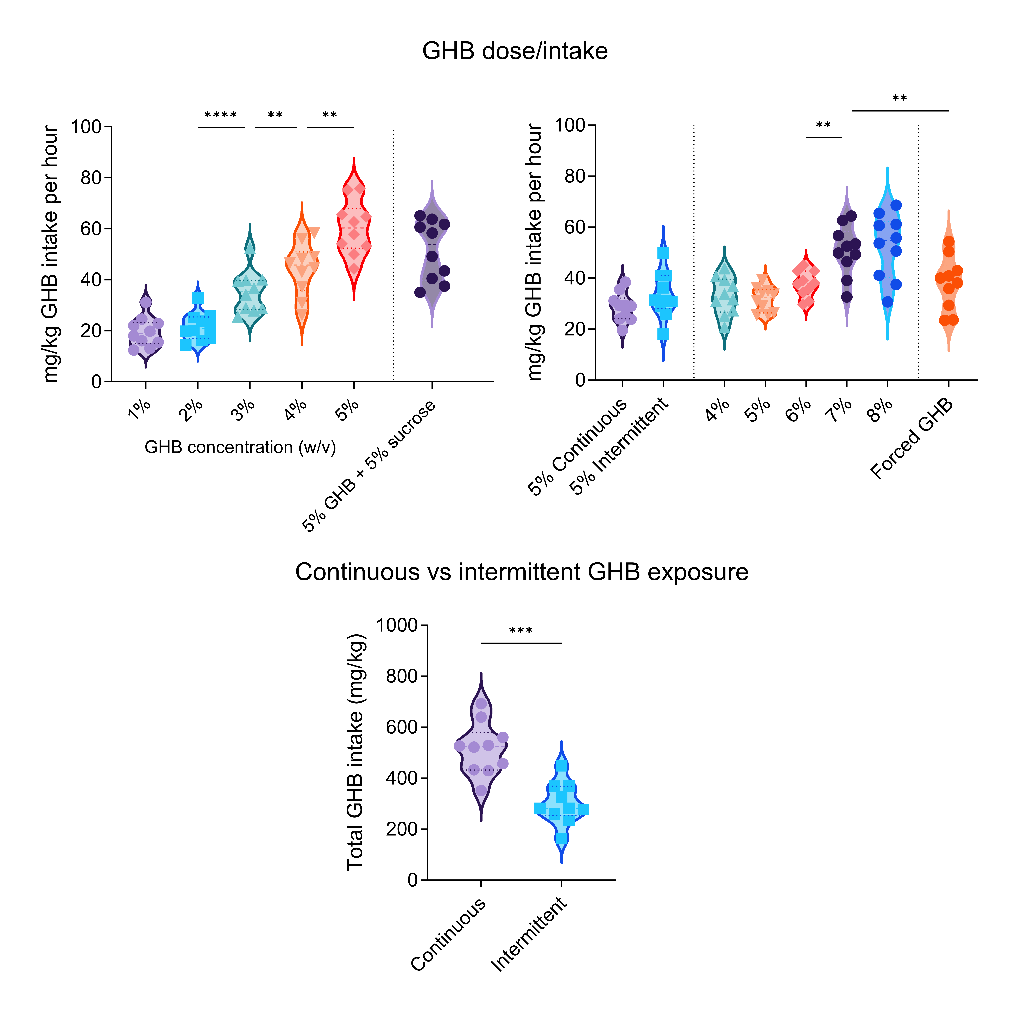


c

**Supplemental Figure 1** Determining optimal GHB self-administration parameters. a) GHB concentration was increased from 1% to 5% GHB (w/v). Intake of 5% GHB (w/v) was subsequently compared to a 5% GHB + 5% sucrose solution. b) Average intake of 5% GHB (w/v) under continuous access was compared with 5% GHB (w/v) intermittent access (GHB available on Mondays, Wednesdays and Fridays). Hereafter, GHB concentration was increased from 4% to 8% GHB (w/v), followed by assessment of GHB intake in absence of water for 3 hours per day (forced GHB). c) Total GHB intake over five days of continuous access was compared to total GHB intake under intermittent access. No escalation in GHB intake was observed in the intermittent or continuous access paradigm. For every condition except “continuous vs intermittent” and “forced GHB”, GHB was available for 6 hours per day on Mondays and Tuesdays (hourly monitoring of intake and GHB overdosing), after which GHB was present 24 hours per day on Wednesdays, Thursdays and Fridays. n = 10. ** = p < 0.01 Bonferroni multiple comparison, *** = p < 0.001 two-sample t-test, **** = p < 0.0001 Bonferroni multiple comparison

**
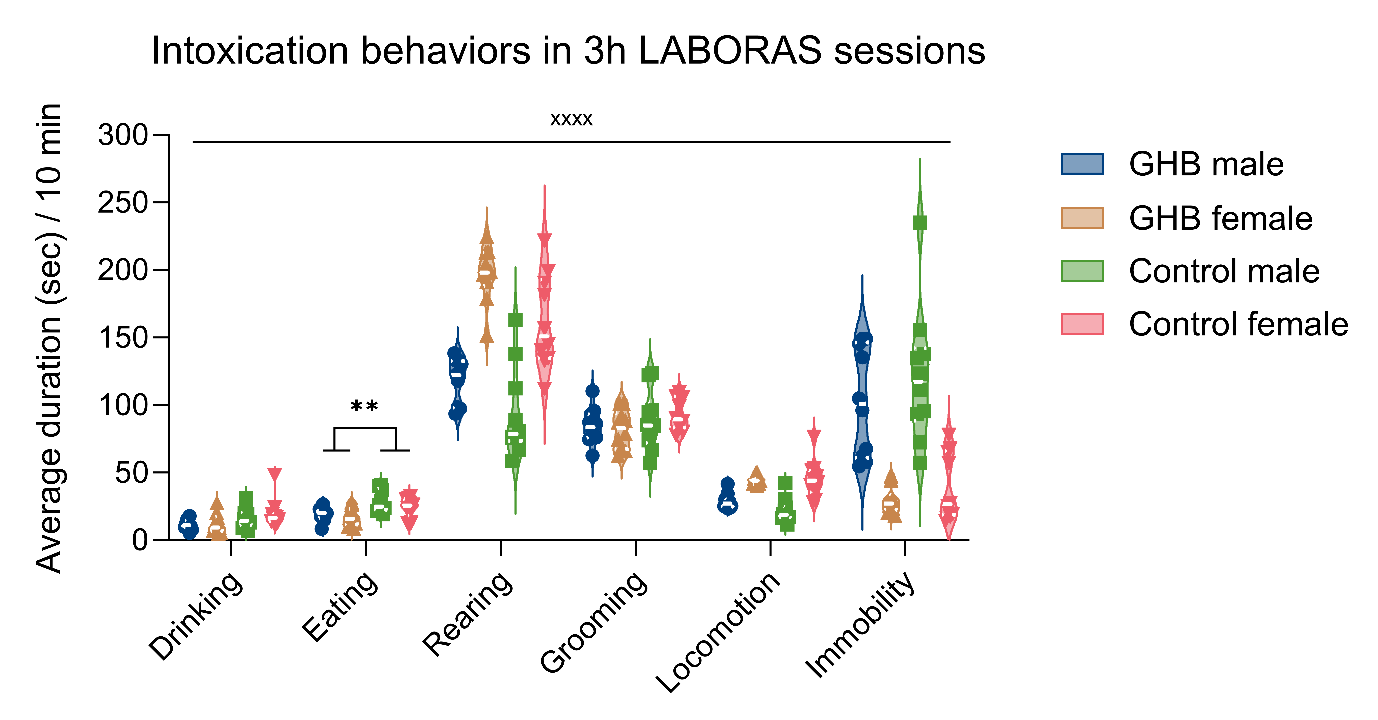
**

**Supplemental Figure 2** Three-hour behavioral assessment during single-housed LABORAS measurements with access to GHB or water. Session 1 (after one month of home-cage GHB self-administration) and session 2 (after two months of home-cage GHB self-administration) are averaged in this figure. n = 10 per group. xxxx = p < 0.0001 three-way ANOVA, behavior x drug interaction; ** = p < 0.01 Bonferroni multiple comparison

**
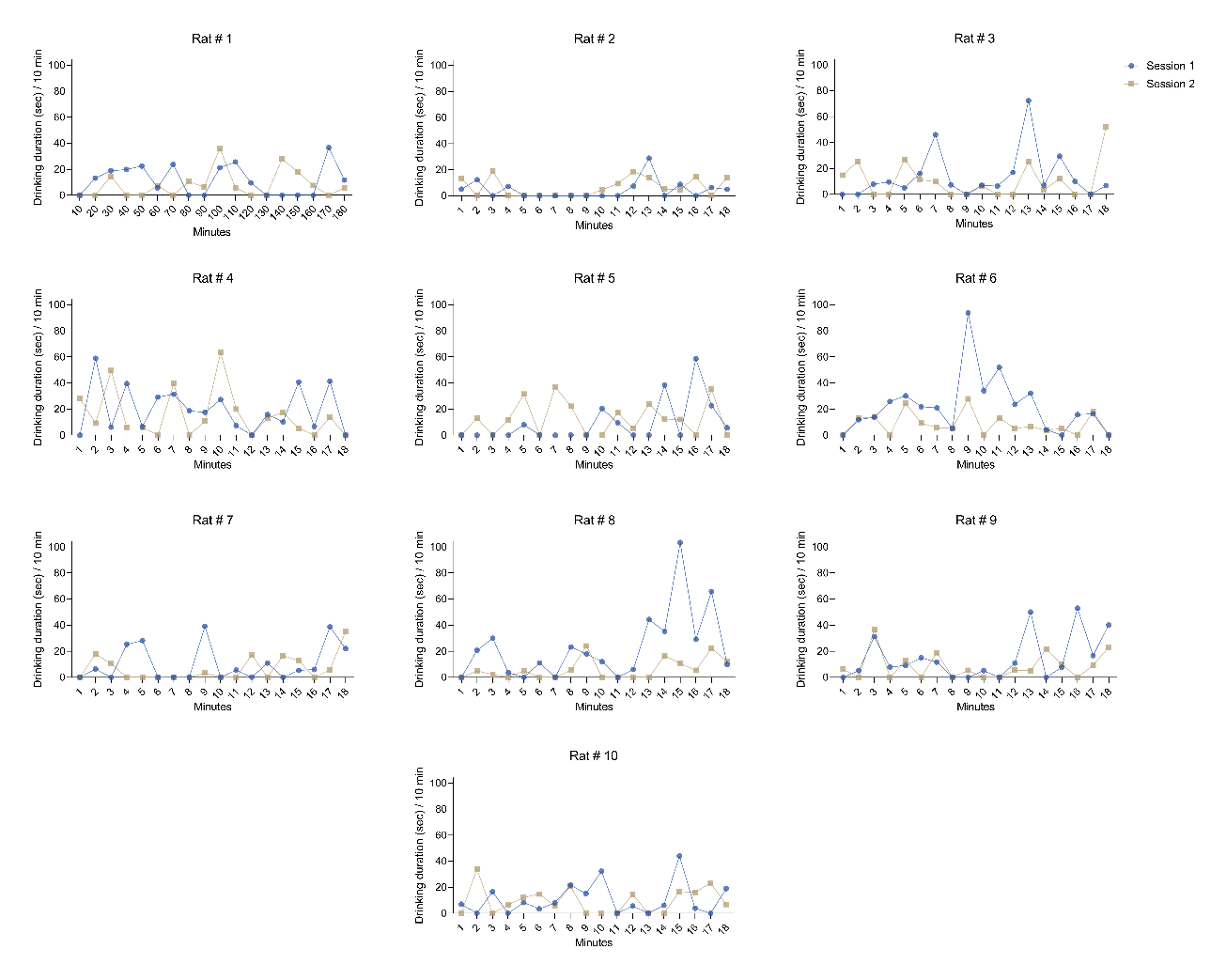

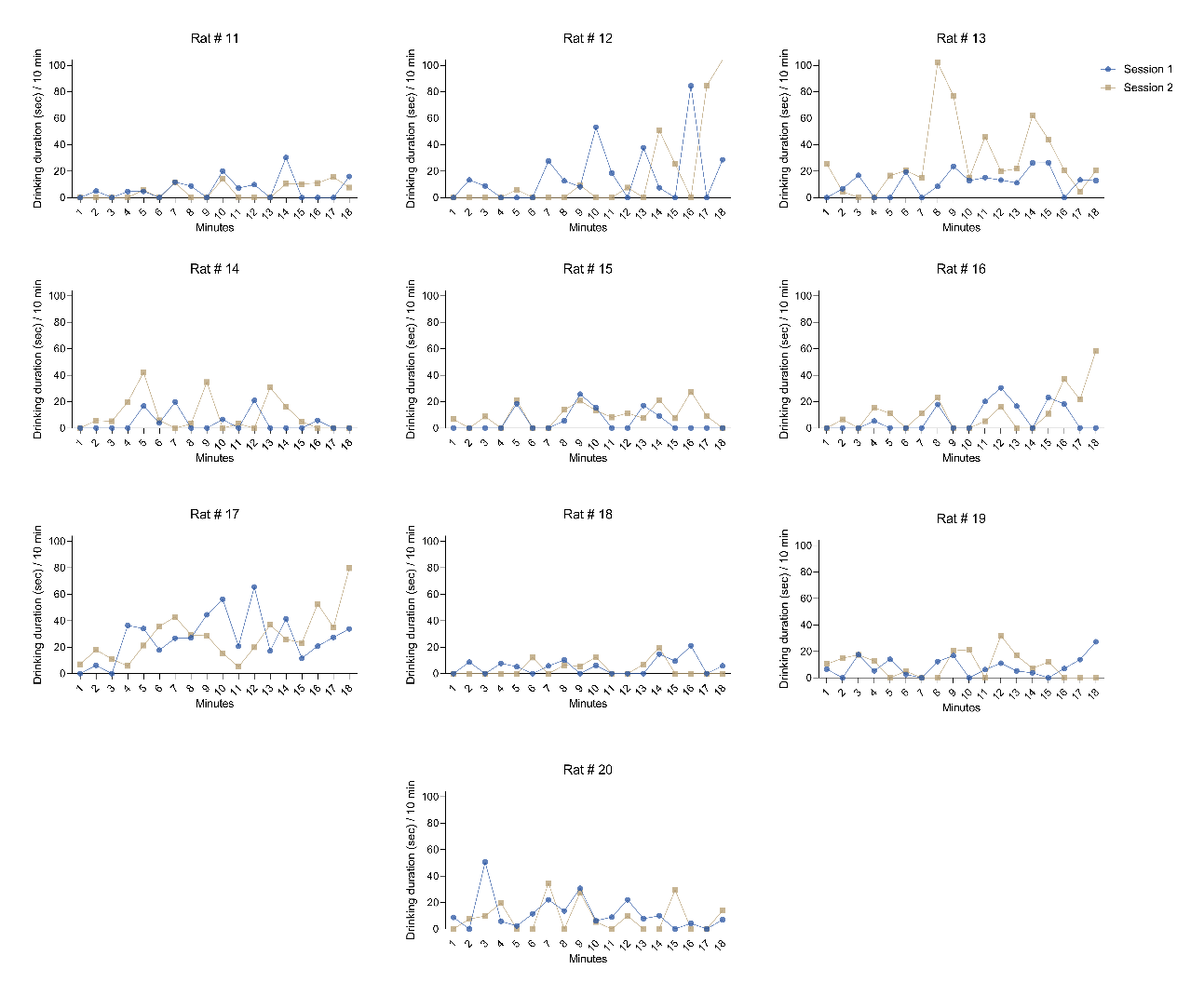
**

a

b

**Supplemental Figure 3** Individual duration of GHB consumption during three-hour LABORAS sessions. A) Consumption of GHB in male rats divided in 10-minute bins. B) Consumption of GHB in female rats divided in 10-minute bins

**
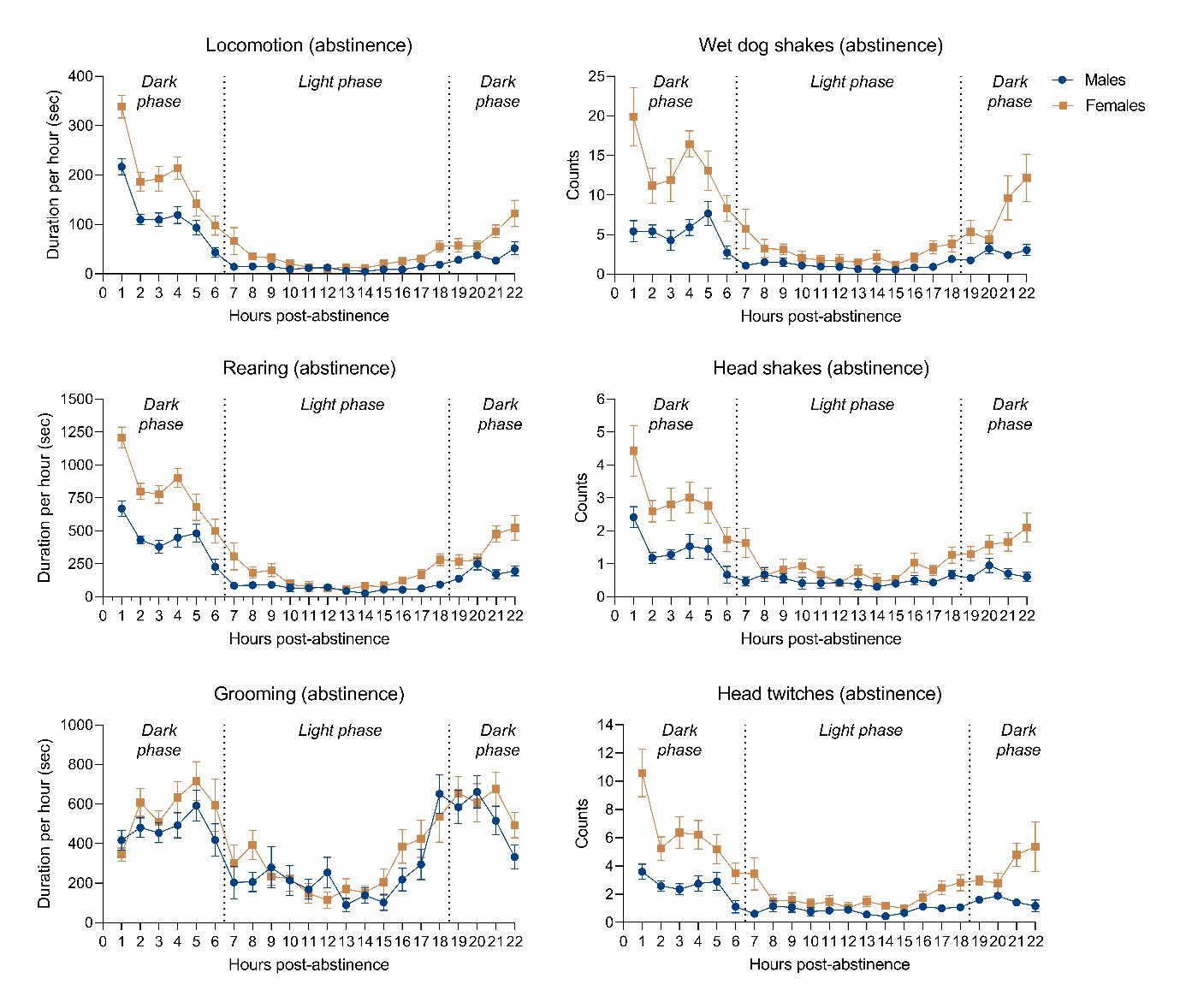
**

e

d

f

a

b

c

**Supplemental Figure 4** Withdrawal behaviors during 22-hour LABORAS sessions following the three-month GHB self-administration period. Session 1 (after one month of home-cage GHB self-administration) and session 2 (after two months of home-cage GHB self-administration) were averaged in this figure. The dark phase corresponds to the rat’s active phase, the light phase corresponds to the rat’s inactive phase. Male n = 10, female n = 10

**
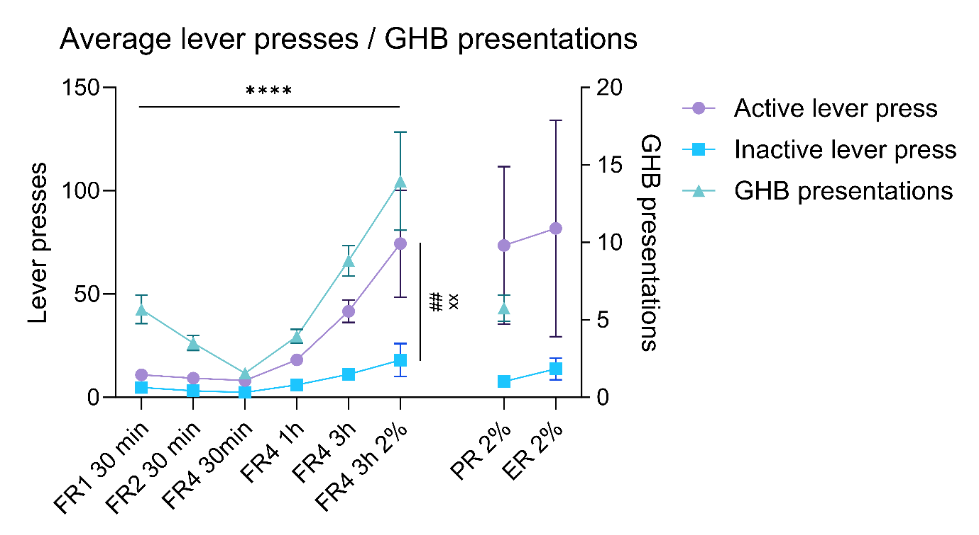
**

**Supplemental Figure 5** GHB presentations and active / inactive lever presses during different operant sessions. All values are shown as average ± SEM. n = 12. **** = p < 0.0001 two-way ANOVA main effect of session type; ## = p < 0.01 main effect of lever type; xx = p < 0.01 session type x lever type interaction. FR = fixed ratio, PR = progressive ratio, NR = non-reinforced


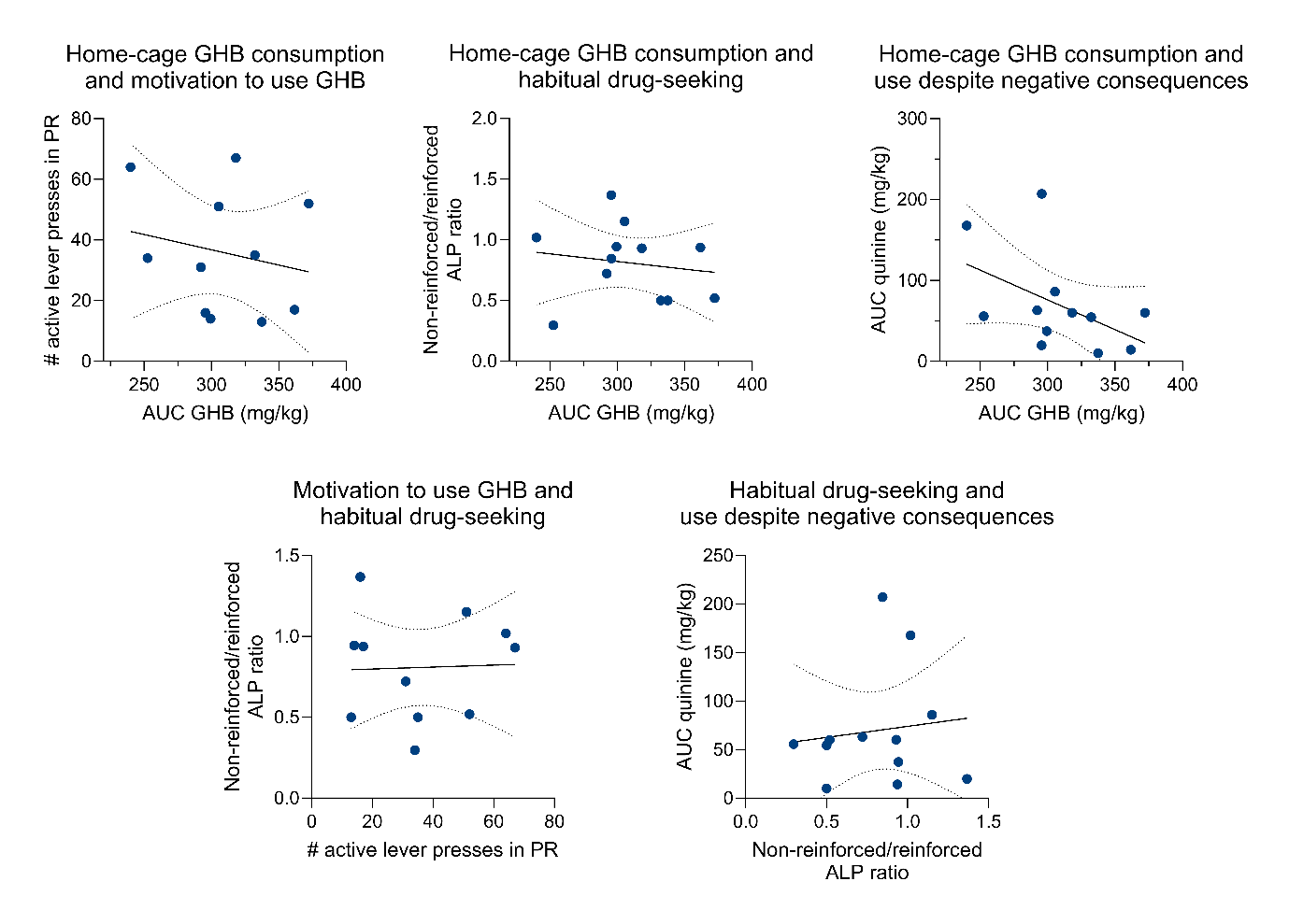


d

e

c

b

a

**Supplemental Figure 6** a) Correlation between home-cage GHB consumption and motivation to use GHB. GHB intake is represented as the area-under-curve (AUC) for the 12-week period of home-cage self-administration, and motivation to use GHB is represented as the total number of active lever presses in the 3h PR test in the operant cage. b) Correlation between home-cage GHB consumption and habitual drug-seeking. Habitual drug-seeking is represented as the ratio between active lever presses during a non-reinforced session, and active lever presses during a session where active lever presses were reinforced with GHB. c) Correlation between home-cage GHB consumption and use despite negative consequences. Use despite negative consequences is represented as the AUC for the quinine-adulterated GHB operant sessions. d) Correlation between motivation to use GHB and habitual drug-seeking. e) Correlation between habitual drug-seeking and quinine-adulterated GHB consumption. n = 11 – 12. Data points represent individual animals. Black line represents the regression line, curved dotted lines represent the 95% confidence interval.


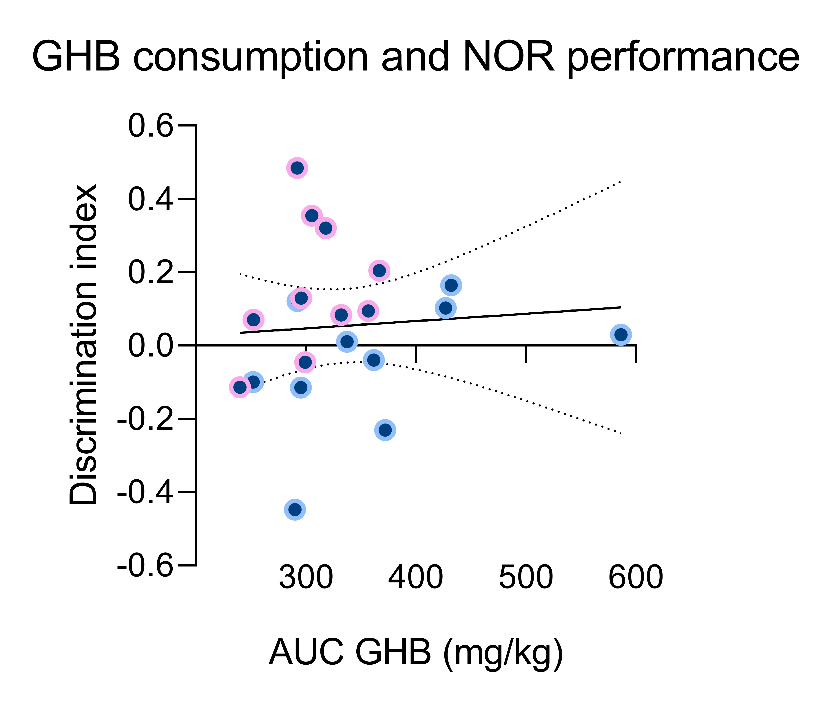


**Supplemental Figure 7** The effect of baseline home-cage GHB intake on novel object recognition performance. Discrimination index is calculated as the difference between the time exploring the novel object and the old object, divided by the total exploration time. GHB intake is expressed as the area-under-curve (AUC) for the 12-week period of home-cage self-administration. Dotted lines represent 95% confidence interval. Blue = males, pink = females


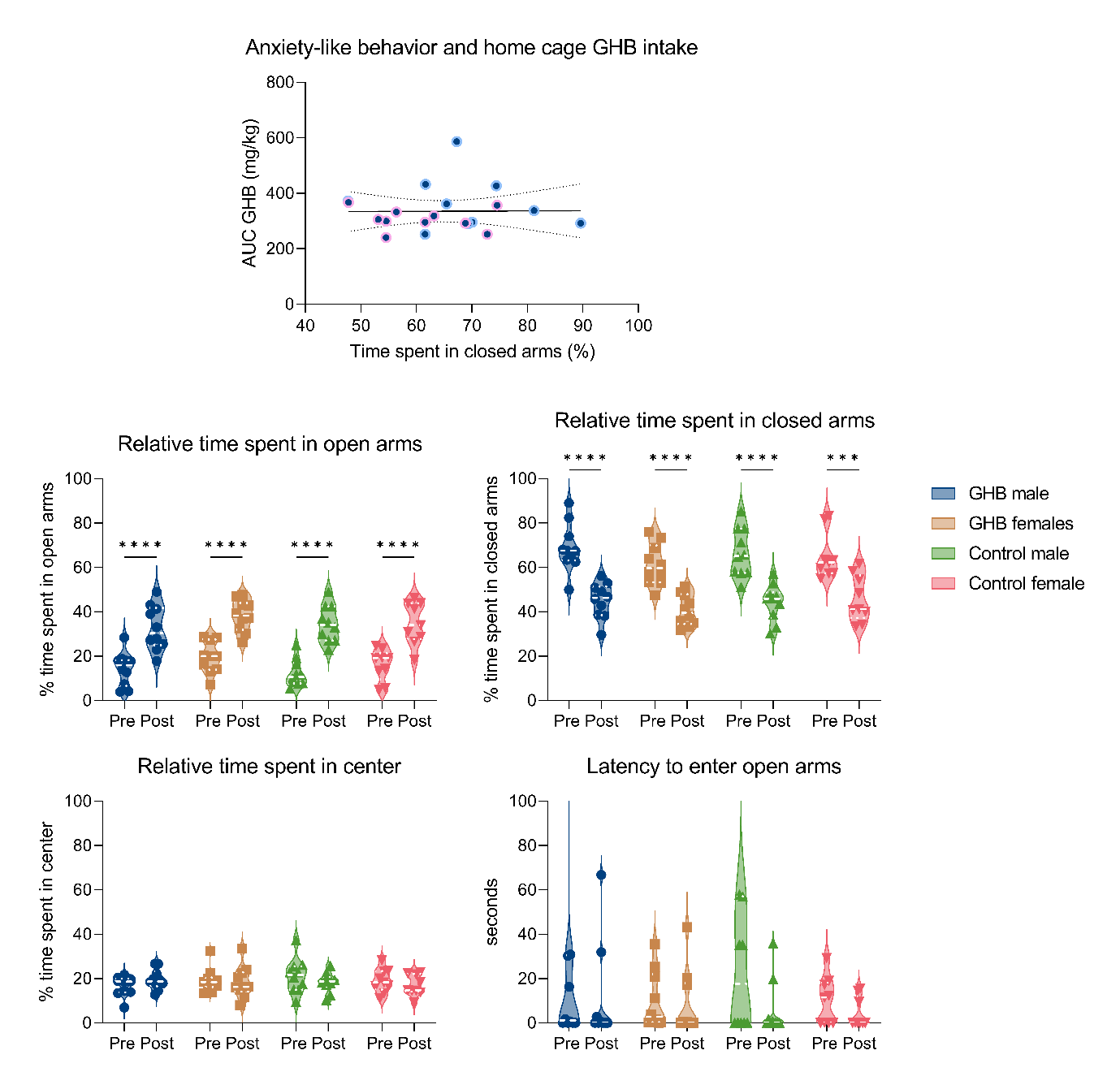


b

a

c

e

d

**Supplemental Figure 8** a) The effect of baseline anxiety-like behavior on home-cage GHB intake. GHB intake is expressed as the area-under-curve (AUC) for the 12-week period of home-cage self-administration. Anxiety-like behavior is expressed as relative time spent in closed arms in the elevated-plus maze. b) Effect of home-cage GHB or water intake and abstinence on time spent in the open arms. c) Effect of home-cage GHB or water intake and abstinence on time spent in the closed arms. d) Effect of home-cage GHB or water intake and abstinence on time spent in the center of the elevated plus maze. e) Effect of home-cage GHB or water intake and abstinence on latency to enter the open arms. GHB n = 20, control n = 19. *** = p < 0.001, **** = p < 0.0001 Bonferroni multiple comparison
